# Epigenetic mechanisms in hepatitis B virus-associated hepatocellular carcinoma

**DOI:** 10.20517/2394-5079.2020.83

**Published:** 2021-02-03

**Authors:** Ourania Andrisani

**Affiliations:** Department of Basic Medical Sciences and Purdue Center for Cancer Research, Purdue University, West Lafayette, IN 47907, USA.

**Keywords:** Hepatitis B virus, hepatocellular carcinoma, epigenetics, DNA methylation, chromatin/histone modifications, lncRNA, miRNA

## Abstract

Chronic infection of the liver by the hepatitis B virus (HBV) is associated with increased risk for developing hepatocellular carcinoma (HCC). A multitude of studies have investigated the mechanism of liver cancer pathogenesis due to chronic HBV infection. Chronic inflammation, expression of specific viral proteins such as HBx, the integration site of the viral genome into the host genome, and the viral genotype, are key players contributing to HCC pathogenesis. In addition, the genetic background of the host and exposure to environmental carcinogens are also predisposing parameters in hepatocarcinogenesis. Despite the plethora of studies, the molecular mechanism of HCC pathogenesis remains incompletely understood. In this review, the focus is on epigenetic mechanisms involved in the pathogenesis of HBV-associated HCC. Epigenetic mechanisms are dynamic molecular processes that regulate gene expression without altering the host DNA, acting by modifying the host chromatin structure via covalent post-translational histone modifications, changing the DNA methylation status, expression of non-coding RNAs such as microRNAs and long noncoding RNAs, and altering the spatial, 3-D organization of the chromatin of the virus-infected cell. Herein, studies are described that provide evidence in support of deregulation of epigenetic mechanisms in the HBV-infected/-replicating hepatocyte and their contribution to hepatocyte transformation. In contrast to genetic mutations which are permanent, epigenetic alterations are dynamic and reversible. Accordingly, the identification of essential molecular epigenetic targets involved in HBV-mediated HCC pathogenesis offers the opportunity for the design and development of novel epigenetic therapeutic approaches.

## INTRODUCTION

In the last decade, the emergence of whole genome sequencing approaches has generated evidence that cancer pathogenesis involves genetic mutations of cancer driver genes and also epigenetic deregulation of critical cellular processes^[1]^. Epigenetic deregulation/aberration in cancer targets every aspect of chromatin biology, including post-translational modifications of histones, DNA methylation, chromatin remodeling, non-coding RNAs, and alterations of 3-D chromatin architecture.

Epigenetic modifications are heritable changes in gene expression that do not involve changes in the DNA sequence and they include the following: (1) DNA methylation by DNA methyltransferases (DNMTs). Methylation of cytosine residues generates 5-methylcytosine, linked to gene silencing^[2]^, while conversely, removal of DNA methylation via Ten-eleven-translocation (TET) dioxygenase-mediated oxidation of 5-methylcytosine is associated with gene activation^[3]^; (2) histone modifications linked to gene activation or gene repression, referred to as the “histone code”. These modifications include methylation, acetylation, phosphorylation, and ubiquitination at various sites of the N-terminal of histone tails^[4]^; (3) re-structuring of the nucleosome, the basic unit of chromatin organization, by ATP-dependent chromatin remodeling complexes during transcription^[5]^; (4) altered expression of long non-coding RNAs (lncRNA) shown to participate in vital molecular processes of chromatin organization, transcription, and RNA processing^[6]^, and of microRNAs (miRNAs) that regulate gene expression post-transcriptionally^[7]^; and (5) alterations in the spatial 3-D architecture of chromatin in the interphase nucleus.

Nearly 15% of human cancers are virus-induced^[8]^. Key principles regarding human oncogenic viruses such as hepatitis B virus (HBV), include the following: (1) oncoviruses encode oncoproteins essential for viral replication; (2) these viral oncoproteins deregulate key cellular networks, which provides the virus a biosynthetic advantage and also alters host cell growth, thereby contributing to oncogenic transformation; (3) cancer development occurs after persistent infection; and (4) environmental carcinogens, host genetic mutations, and immune responses are additional players in virus-induced cancers. Importantly, virus/host interactions influence directly or indirectly the epigenetic chromatin landscape of the infected cell^[9]^. Since most epigenetic mechanisms can be reversed, unlike genetic mutations, the identification of essential epigenetic targets offers the opportunity for the design of novel epigenetic therapies. This review focuses on HBV-mediated epigenetic events and their role in liver cancer pathogenesis.

## HBV LIFE CYCLE

HBV is a non-cytopathic hepadnavirus, containing a partially double-stranded genome. The 3.2-kb HBV genome is compact, generating four overlapping RNAs encoding the genes of viral reverse transcriptase/polymerase (P), HBx (X), core antigen (C) and surface/envelope antigen (HBsAg). HBV uses the sodium taurocholate co-transporting polypeptide (NTCP) receptor to attach to hepatocytes^[10,11]^. After entry, HBV nucleocapsids transport HBV DNA to the nucleus, where the relaxed circular DNA is converted into covalently closed circular DNA (cccDNA). cccDNA forms chromatin-like structure, referred to as the viral minichromosome, acting as template for transcription of all viral RNAs (0.7, 2.1, 2.4 and 3.5 kb). The mRNA transcripts are exported to the cytoplasm and used for translation of the viral proteins. The 3.5-kb pregenomic RNA also functions as template for viral replication occurring within nucleocapsids, formed by the core protein in the cytoplasm. HBV nucleocapsids are enveloped during their passage through endoplasmic reticulum (ER)-associated intracellular compartments, and secreted from the hepatocyte^[12,13]^.

## CHRONIC HEPATITIS B AND HEPATOCELLULAR CARCINOMA

According to the World Health Organization (WHO), more than 250 million people globally are chronically infected with HBV, and nearly 800,000 patients die every year due to HBV-mediated complications of liver function. It is well established that chronic HBV infection is associated with increased risk for liver cancer^[14]^. Hepatocellular carcinoma (HCC) develops by progressing from the preneoplastic to the cancerous phase. Chronic inflammation characterized by repeated cycles of apoptosis, necrosis, and regeneration is an important contributor to hepatocarcinogenesis^[12,15–17]^. Additionally, cell intrinsic mechanisms contribute to HBV-mediated hepatocarcinogenesis. These include: (1) interaction of the HBsAg proteins with the ER, inducing ER and oxidative stress^[18]^, stimulating growth and survival-signaling pathways, and causing mutations by the generation of free radicals^[19]^; (2) integration of the HBV genome in the host genome inducing DNA deletions^[20]^, or by insertion in proximity to cancer-relevant genes, including cyclin A, telomerase reverse transcriptase (TERT), platelet-derived growth factor receptor-beta (PDGFR-beta), mitogen-activated protein kinase 1 (MAPK1), and others^[21–23]^; (3) activation of cellular mitogenic signaling cascades by the essential viral HBx protein^[24–29]^, resulting in the activation of the downstream transcription factors NF-kB, AP-1, AP-2, c-EBP, and ATF/CREB^[30–33]^, thereby altering the expression of cellular growth-control genes. Significantly, HBx/c-Myc bi-transgenic mice expressing c-Myc under the control of the woodchuck hepatitis virus (WHV) regulatory elements, exhibit accelerated formation of liver tumors^[34]^, demonstrating the cofactor role of HBx in HBV-mediated hepatocarcinogenesis; and (4) infection by distinct HBV genotypes, associated with poor prognosis HBV-related liver cancer^[35]^. For example, the HBV F1b genotype containing specific mutations (T1938C & A2051C) in the coding region of HBc is associated with increased HCC risk in chronically HBV infected Alaskan Natives (indigenous peoples)^[36]^. HBc could also be endowed with oncogenic properties and collaborate with HBx in the pathogenic process, since both HBx^[37,38]^ and HBc^[39]^ are nuclear proteins found in association with the HBV minichromosome.

## MUTATIONAL LANDSCAPE OF HBV-RELATED HCC

HCCs of different etiologies, including chronic infection by HBV, have been used to determine the genetic landscape of HCC, by whole genome and exome sequencing^[40–44]^. The data show that overall, HCCs have multiple mutations with significant incidence, thereby contrasting other solid tumors, e.g., pancreatic and lung cancers, characterized by the presence of the single driver RasV12 mutation. HBV-mediated HCCs were reported to be associated with single nucleotide polymorphisms (SNPs) of various genes^[45]^, suggesting that host genetic factors contribute to HCC susceptibility^[46]^. Furthermore, tumor tissues from HBV infected patients displayed TERT promoter mutations and TERT gene amplification, which rarely co-occurred with HBV integration in the TERT locus^[40]^, while highly rearranged copy number profiles have been found in HBV-related liver tumors^[41]^. HBV-mediated HCCs are also characterized by mutations in the tumor suppressor p53 (TP53), WNT pathway (APC, AXIN1, CTNNB1), telomere maintenance (TERT), and epigenetic enzymes (ARID1A, ARID2, MLL4)^[39,40]^. It is not understood why HBV-associated HCCs exhibit a prevalence of mutations in these genes, in comparison to HCCs of other etiologies. More importantly, not all HBV-related liver tumors contain these genetic alterations or tumor driver mutations^[47]^, raising the question of what other mechanisms alter the cellular program of the HBV-infected hepatocyte toward oncogenic transformation.

## 3-D ARCHITECTURE OF CHROMATIN

Chromosomes occupy specific positions in the interphase nucleus, called chromosome territories, relative to other nuclear structures such as the nuclear lamina, paraspeckles and promyelocytic leukemia nuclear bodies (NBs). Within chromosome territories, the chromatin exhibits a hierarchy in folding, ranging from chromosomal compartments, to topologically associating domains (TADs), chromatin loops, and enhancer-promoter contacts^[48]^. Disruption of enhancer-promoter contacts and alteration of nuclear subcompartments play important roles in diseases, including cancer^[48]^. Many viruses enter into the nucleus as part of their biosynthetic pathway, including HBV, Epstein-Barr virus, human lymphotropic virus type 1 (HTLV1), human papillomavirus (HPV), and human immunodeficiency virus (HIV-1), among others^[49]^. Thus, one must consider the implications of the entry of the viral nucleic acid into the nucleus and its effect on the 3-D nuclear architecture of the host genome.

## 3-D ARCHITECTURE OF CHROMATIN AND HBV INFECTION

Recent studies^[50]^ have investigated how the HBV minichromosome positions itself relative to the chromatin of the infected cell, employing Hi-C and viral DNA capture (CHi-C) approaches. Specifically, the interaction of the HBV cccDNA/minichromosome^[51]^ with the host genome was investigated during a 7-day infection of primary human hepatocytes (PHH). cccDNA serves as template for transcription of viral RNAs using cellular transcription factors. The rate of cccDNA transcription is influenced by the state of the chromatin modifications of the cccDNA/minichromosome^[52]^. It has been shown^[50]^ that the HBV cccDNA positions preferentially at CpG islands (CGIs) of active genes, and this cccDNA association results in deregulation of cellular gene expression. Interestingly, one of the most highly contacted genes identified by these experiments is the gene encoding lncRNA HOX transcript antisense RNA (HOTAIR)^[50]^. HOTAIR is upregulated in HBV-related HCCs^[53]^ but not during a 7-day infection of PHHs^[50]^. Thus, the consequence of the association of the viral minichromosome with the host chromatin, during the course of chronic HBV infection, in the deregulation of cellular gene expression and oncogenic transformation, remains to be elucidated.

## EPIGENETIC MECHANISMS IN HBV-ASSOCIATED HCC

Toward understanding the mechanism of HBV-mediated hepatocarcinogenesis and considering the lack of an amenable animal model that recapitulates the human disease, an alternative approach is to combine and interpret *in vitro* mechanistic studies with data derived from analyses of human HBV-associated liver tumors. Here, I first describe results derived from analyses of human liver tumors, generated by the transcriptomic studies of Boyault *et al.*^[54]^. Using unsupervised transcriptome analysis, 6 groups of liver tumors (G1-G6) were identified. Two of these groups, G1 and G2, comprised HBV-related HCCs. G1 tumors had low copy numbers of HBV and exhibited overexpression of fetal liver genes (AFP and SOX9), and parentally imprinted genes including insulin-like growth factor 2 (IGF2), H19, and paternally expressed genes 3 and 10 (PEG3 and PEG10). G2 tumors included HCCs exhibiting high copy numbers of HBV and containing mutations in PIK3CA and TP53.

Reactivation of imprinted genes, as observed in the G1 HBV-associated HCCs^[54]^, is mediated by epigenetic mechanisms that involve loss of DNA methylation or loss of the silencing trimethylation of lysine 27 of histone3 (H3K27me3)^[55]^. Interestingly, recent studies demonstrated that during normal liver regeneration, epigenetic mechanisms redistribute/remove the silencing H3K27me3 from the promoters of pro-regenerative genes, allowing their expression^[56]^. H3K27me3 is mediated by the polycomb repressive complex 2 (PRC2), comprised of the essential core subunits EZH2, possessing the methyltransferase activity, SUZ12, and EED^[57]^. Human tumors exhibiting loss of imprinting lack of association of the silencing H3K27me3 with the reactivated genes^[58]^. The absence of this silencing modification (H3K27me3) from the reactivated genes is due to reduction in the level of SUZ12 protein, which in turn, results in reduced level of the PRC2 complex^[58]^. This mechanism of genomic imprinting based on reduced H3K27me3 is independent of DNA methylation^[59]^. Together, these observations lead to the conclusion that deregulation of epigenetic mechanisms mediate the expression of the imprinted genes observed in the G1 group of HBV-related tumors^[54]^. This raises the question of how HBV infection deregulates this epigenetic mechanism.

## PRC2 AND HBV-RELATED HCC

Downregulation of the PRC2 subunit SUZ12 was initially identified by a siRNA library screen, performed in a model cell line that expresses HBx conditionally^[60]^. Based on this screen, knockdown of SUZ12 rescued HBx-expressing cells from DNA damage-induced apoptosis^[60]^, suggesting that the loss of SUZ12 imparts a growth advantage to the HBx-expressing cell. In this cell line, HBx also activates cellular mitotic polo-like-kinase (PLK1)^[61]^, a pro-viral host factor required for HBV replication^[62]^. Interestingly, liver tumors from woodchucks chronically infected with the woodchuck hepatitis virus and liver tumors from HBx/c-Myc bitransgenic mice^[34]^ both exhibited an inverse relationship between PLK1 and SUZ12, namely high PLK1 *vs*. low SUZ12 protein levels^[63]^. On the basis of this observation, subsequent studies demonstrated that activated PLK1 phosphorylates SUZ12, resulting in proteasomal degradation of SUZ12^[53]^. This process of SUZ12 degradation requires lncRNA HOTAIR^[53]^. It is intriguing, that the chromatin of the HOTAIR gene is highly contacted by the cccDNA during HBV infection, as described by the study of Moreau *et al*.^[50]^. Whether chronic HBV infection contributes to upregulated HOTAIR expression is presently unknown.

**Figure 1.**
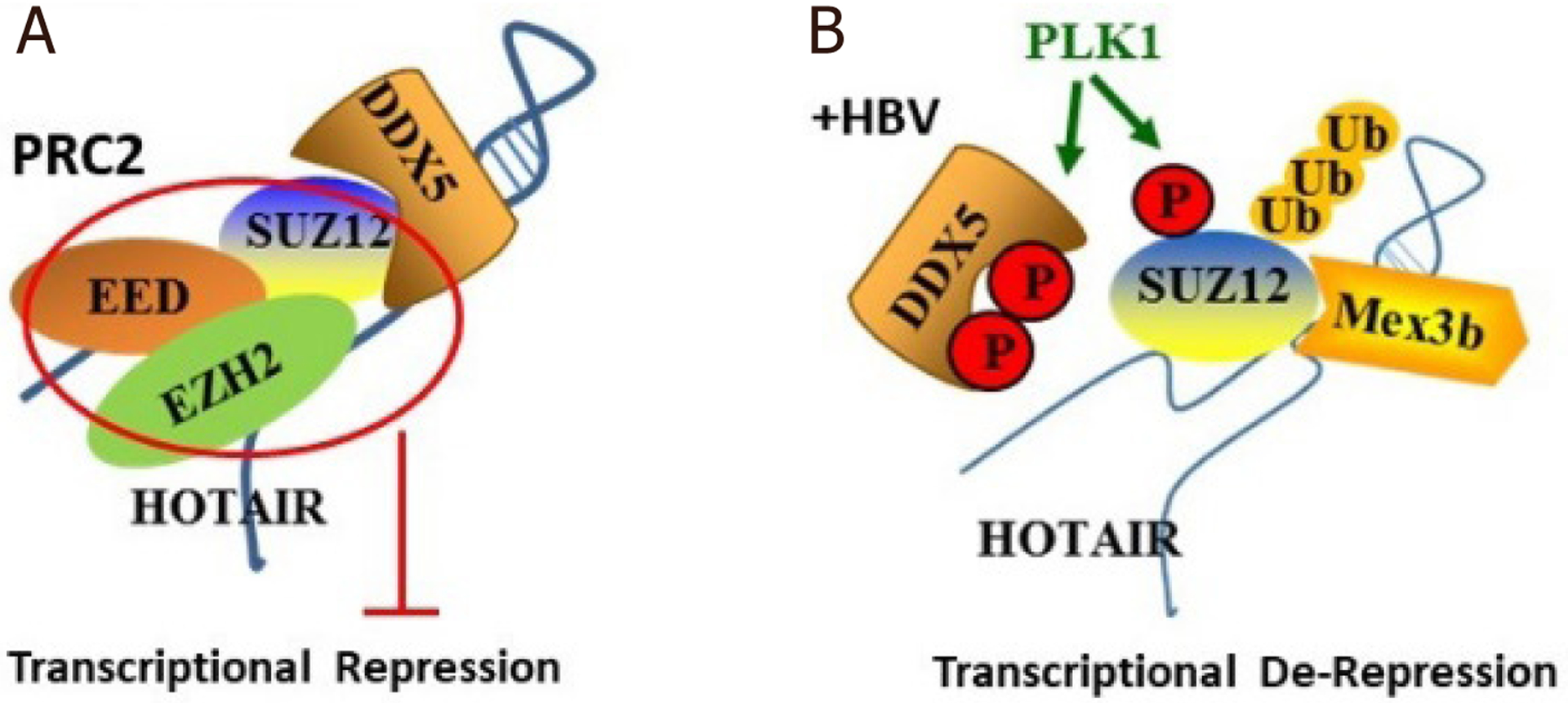
Deregulation of the epigenetic PRC2 complex by HBV infection. A: Diagram depicts the PRC2 complex, comprised of the core subunits EZH2, EED and SUZ12, in interaction with RNA helicase DDX5 and lncRNA HOTAIR; B: working model of how HBV infection disrupts the transcriptionally silencing PRC2. HBV infection promotes activation of PLK1^[61,62]^, which phosphorylates SUZ12. E3 ligase Mex3b ubiquitinates SUZ12, leading to SUZ12 proteasomal degradation^[64]^. The functional significance of the phosphorylation of DDX5 by PLK1 remains to be determined (Rahman and Andrisani, unpublished results)

The noncoding RNA HOTAIR serves as a scaffold structure in this process that leads to proteasomal degradation of SUZ12 [Figure 1]. HOTAIR interacts with the PRC2 complex, and two additional RNA-binding proteins, E3 ligase Mex3b and RNA helicase DDX5. Mex3b ubiquitinates SUZ12, inducing its degradation, whereas DDX5 prevents SUZ12 ubiquitination and degradation, thereby stabilizing the repressive function of PRC2^[64]^. Interestingly, poor prognosis HBV-related liver tumors exhibit downregulation of DDX5^[64]^.

The downregulation of DDX5 results in downregulation of SUZ12, reduction of the repressive PRC2 complex, and reactivation of the PRC2 target genes EpCAM and pluripotency genes^[64]^. EpCAM, a transmembrane glycoprotein involved in cell signaling^[65,66]^, is highly expressed in carcinomas, tumor-initiating cells, tissue progenitor cells, embryonic and adult stem cells, but at lower levels in non-transformed epithelia^[67]^. EpCAM is expressed in hepatic progenitors^[68]^ and hepatic cancer stem cells (hCSCs)^[69]^. Our recent *in vitro* studies showed that the downregulation of DDX5 results in the activation of Wnt signaling, and appearance of features characteristic of hCSCs. Such features include hepatosphere formation, expression of EpCAM, resistance to chemotherapeutic agents, and deregulation of genes exerting an effect on Wnt signaling activation^[70]^. Specifically, the positive regulator of Wnt signaling *disheveled 1*, DVL1, is upregulated in HCCs with low DDX5 mRNA levels, on the basis of the analysis of liver tumors available through The Cancer Genome Atlas (TCGA), and LIMORE cell lines^[47]^ derived from HBV-related liver tumors^[70]^.

On the other hand, EZH2, the methyltransferase component of PRC2, is upregulated in many cancer types, including HBV-related HCCs^[71]^. EZH2 can function independently of the other PRC2 subunits, methylating non-histone proteins^[72]^ such as STAT3, which results in enhanced STAT3 activation, as has been observed in glioblastoma^[73]^. PRC2-independent functions for EZH2 have also been identified in prostate cancer, where EZH2 acts as co-activator of the androgen receptor^[72]^. EZH2 and its associated PRC2 complex are the most significantly deregulated epigenetic regulators in primary HCC^[74]^. Increased expression of EZH2 in HCC results in the suppression of miRNAs, modulating cell motility and metastasis-related pathways^[74]^, and in the activation of Wnt signaling by silencing Wnt antagonists^[75]^. Thus, Wnt signaling, one of the key pathways that contribute to the expression of pluripotency genes and a progenitor-like phenotype^[45]^, is activated by the downregulation of the RNA helicase DDX5^[70]^ and/or by the enhanced expression of EZH2, suppressing the expression of Wnt antagonists^[75]^. EZH2 also interacts with noncoding RNAs, including lncRNA DLEU2 to sustain cccDNA transcription and transcription of cancer relevant genes^[76]^. It is important to note that in HBV-related HCCs, the activation of Wnt signaling due to CTNNB1 activating mutations and/or inactivating mutations in AXIN or APC is infrequent in comparison to other HCC etiologies^[77]^. Moreover, HCCs with CTNNB1 mutations have better overall prognosis^[45,54]^.

## NONCODING RNAS IN HBV-RELATED HCC

In addition to protein coding genes, nearly 75% of the human genome encodes genes for non-protein coding RNAs. These include long noncoding RNAs (lncRNAs) > 200 nucleotides (nt), and noncoding RNAs < 200 nt that also include miRNAs (miRs). Both classes of noncoding RNAs function epigenetically in regulating gene expression via distinct mechanisms. Consequently, their deregulated expression has important implications in diseases including cancer^[78,79]^. miRs downregulate gene expression by targeting either mRNA stability or by inhibiting the translation of genes functioning in related cascades^[79]^. The mechanism of lncRNA-mediated epigenetic regulation is more complex^[6]^. LncRNAs can exert effects on chromatin organization, transcription, post-transcriptional modifications, signal transduction, and nuclear organization serving as architectural scaffolds; for example, lncRNAs MALAT1^[80]^ and NEAT1^[81]^ function as structural scaffolds of nuclear speckles and paraspeckles, respectively^[6]^. Several noncoding RNAs shown to be involved in HBV-mediated hepatocarcinogenesis are listed in Table 1 and reviewed in Guerrieri F^[82]^.

LncRNA highly upregulated in liver cancer (HULC), up-regulated in liver cancer, is involved in activation by phosphorylation of the transcription factor CREB^[83]^, mediated by protein kinase A (PKA)^[84]^. Activated CREB interacts with the histone acetyl transferase CBP/p300^[84]^, thereby maintaining transcriptionally active chromatin of CREB-responsive promoters. HULC functions as a “sponge” in sequestering miR-372, a miRNA that downregulates the expression of the catalytic subunit of PKA^[83]^. Similarly, HULC can stimulate the transcription of HBx via CREB/CBP/p300 recruitment to the HBV minichromosome^[37]^. Indeed, expression of HBx positively correlated with HULC in clinical HCC tissues^[85]^. Recent studies demonstrated another mechanism leading to increased HBx levels, namely HULC increased cccDNA stability by downregulating APOBEC3B. HULC upregulated miR-539, which targets the 3’UTR of APOBEC3B mRNA^[86]^. Considering the role of HBx in hepatocarcinogenesis, as discussed earlier, and the role of CREB in regulating cell cycle-dependent genes^[87]^, this regulatory network provides an example of how overexpression of HULC regulates both viral biosynthesis and hepatocyte growth control.

LncRNA HOTAIR, Hox transcript antisense RNA, is encoded by the HOXC locus of the HOX gene cluster^[88]^. HOTAIR recruits PRC2 to repress expression of HOXD locus genes^[89]^. HOTAIR also functions as a miRNA “sponge”, thereby regulating de-repression of miRNA targets^[90]^. Most genes repressed by HOTAIR-mediated PRC2 binding are involved in cell signaling, metastasis, and development^[91]^. Increased expression of HOTAIR is observed in liver cancer, and correlates with increased risk of recurrence after hepatectomy and metastasis^[92,93]^. As described earlier [Figure 1], HOTAIR is involved in PLK1-dependent proteasomal degradation of SUZ12^[53]^, acting as scaffold in the antagonistic action of E3 ligase Mex3b *vs*. the PRC2 stabilizing function of RNA helicase DDX5^[64]^. Other lncRNAs reported to be involved in HBV-related liver cancer include lncRNA MALAT1^[94]^ and lncRNA H19^[95,96]^, acting by regulating a set of miRNAs that activate AKT signaling, a mechanism relevant to HBV-related G2 liver tumors^[54]^. Recent studies have reported additional non-coding RNAs having a role in HBV-related HCC, acting via various mechanisms to affect hepatocarcinogenesis^[76,97]^, for example, the small nucleolar RNA SNOR18L5, mediating increased proteolysis of p53^[98]^.

miRNAs deregulated in HBV-related HCCs have been described in the recent comprehensive review by Sartorius *et al.*^[99]^. Herein, I specifically discuss upregulation in HBV-related liver tumors^[100]^ of two proto-oncogenic miRNA clusters, miR106b~25 and miR17~92^[101]^.

miR17~92 is induced by c-Myc^[102]^, and miR106b~25 is encoded within intron 13 of minichromosome maintenance complex component 7 (MCM7)^[103]^. These miR clusters share the same seed sequence on the target mRNAs, and are upregulated in HBV-related^[100,104]^ and WHV-related HCCs^[100]^. Known targets include various tumor suppressor genes such as PTEN, Rb, E2F1, SMAD7^[101]^, and LKB1^[105]^. Recent studies have shown that these two proto-oncogenic miRNA clusters are induced by HBV replication and also target the seed sequence found in 3’UTR of RNA helicase DDX5^[70]^. Thus, HBV infection by increasing the expression of these miRNAs downregulates, among other proteins, the RNA helicase DDX5 [Figure 1]. RNA helicases, including DDX5, are involved in all aspects of RNA metabolism, from transcription, epigenetic regulation, and miRNA processing to mRNA splicing, decay, and translation^[106,107]^. Interestingly, DDX5 was shown to exert antiviral effects on HBV biosynthesis^[64]^. DDX5 knockdown in HBV infected HepG2-NTCP cells increased viral transcription, while the silencing H3K27me3 modification associated with cccDNA was reduced^[64]^. As described earlier [Figure 1], DDX5 interacts with PRC2, epigenetically regulating histone modifications, including modification of the viral minichromosome. Further studies are needed to determine the cellular context of this regulation, for example during the antiviral innate immune response. For the HBV infected hepatocyte, the consequence of DDX5 downregulation is that it imparts cancer stem cell-like properties^[70]^. How DDX5 effects chromatin changes remains to be determined, likely involving interaction with noncoding RNAs^[106]^, and interaction with epigenetic complexes such as PRC2^[64]^ [Figure 1]. Similarly, how DDX5 regulates stemness is incompletely understood. DDX5 has been shown to act as a roadblock of somatic cell reprogramming^[108]^.

On the basis of these functions of DDX5, restoring the protein level of DDX5 in chronically HBV infected hepatocytes could provide therapeutic benefit. Antagomirs (inhibitors) for miR106b~25 and miR17~92 to restore DDX5 will target multiple pathways important for HCC, namely inhibition of HBV replication/biosynthesis, rescue of tumor suppressor genes, and repression of Wnt signaling. Several miRNA-based therapeutic delivery strategies have reached clinical development^[109]^, including lipid-based nanoparticle formulations^[110]^. Hepatocyte-specific deliveries utilize miRNA-conjugation to cholesterol^[111,112]^ and N-acetyl-glucosamine (GalNac), which exhibits high affinity for the asialoglycoprotein receptor expressed in hepatocytes^[109,113]^. Recent studies have also developed folate-linked miRNAs targeting folate receptor-overexpressing cancer cells^[114]^. Our ongoing studies are investigating folate receptor expression in HBV-infected hepatocytes (Andrisani *et al*., unpublished results). Thus, several promising approaches are available to explore antagomir-mediated restoration of DDX5 in chronically infected HBV patients.

## CONCLUSION

Despite the multitude of studies of the hepatitis B virus and its link to liver cancer development, presented in earlier reviews^[115–117]^ and herein, there remains a lot to understand in terms of the mechanism of liver cancer pathogenesis. In this review, I have focused on the presentation of studies that link data derived from human HBV-associated liver tumors to *in vitro* mechanistic results, demonstrating involvement of epigenetic mechanisms in the pathogenesis of HBV-related liver cancer. More efforts must be made by the global clinical and scientific HBV community to develop and make available molecular tools and human tumor samples for generating meaningful data, and for identifying new molecular mechanism-based targets. This is essential for developing novel approaches and therapies to combat HBV-mediated liver cancer.

## Figures and Tables

**Table 1.** Noncoding RNAs in HBV-related HCC noncoding RNA

	Ref.
IncRNA HULC	[83–85]
IncRNA HOTAIR	[53,64]
IncRNA MALAT1	[94]
IncRNA H19	[95,96]
IncRNA HOTTIP	[97]
IncRNA DLEU2	[76]
SNORA18L5	[98]
